# Heat Stress and Feed Restriction Distinctly Affect Performance, Carcass and Meat Yield, Intestinal Integrity, and Inflammatory (Chemo)Cytokines in Broiler Chickens

**DOI:** 10.3389/fphys.2021.707757

**Published:** 2021-07-22

**Authors:** Nima K. Emami, Elizabeth S. Greene, Michael H. Kogut, Sami Dridi

**Affiliations:** ^1^Center of Excellence for Poultry Science, University of Arkansas, Fayetteville, AR, United States; ^2^Southern Plains Agricultural Research Center, USDA-ARS, College Station, TX, United States

**Keywords:** heat stress, broiler chicken, performance, body parts yield, immune response, gut integrity

## Abstract

This study was conducted to distinguish the effects of heat stress (HS) and feed intake (FI) on broiler chicken’s physiological responses. Day-old male Cobb 500 broilers (*n* = 672) were allocated to three treatments: (1) control (CTL): birds raised under normal temperature (23°C) from day 29 to 42; (2) cyclic heat stress (CHS): birds exposed to high temperatures (8 h/day at 35°C; from 9:30 am to 5:30 pm) from day 29 to 42; (3) pair-fed (PF): birds raised under thermoneutral condition but fed the same amount of feed as CHS from day 29 to 42. On day 42, 15 birds/pen were processed, to measure carcass and meat yields. To measure blood parameters and gut integrity (using fluorescein isothiocyanate-dextran), on day 42, CHS birds were sampled before (Pre-CHS) and 2 h after (Post-CHS) the temperature increased. Furthermore, after sampling CTL birds, they were exposed to 2h heat and sampled (acute heat stress, AHS). Data were analyzed using one-way ANOVA (JMP Pro15) and significance between treatments identified by LSD (*P* < 0.05). BW and relative carcass yield were significantly higher in CTL compared to CHS and PF. Compared to CHS, PF had significantly higher BW and lower relative carcass yield. Breast yield was significantly higher for CTL and PF, while leg quarters and wings yield were significantly lower compared to CHS. Gut barrier integrity was significantly altered in Post-CHS and AHS compared to CTL. mRNA abundances of tumor necrosis factor-α, C-C motif chemokine ligand-20, heat shock protein (*HSP*)*-27*, and *HSP70* were significantly higher in Post-CHS and AHS compared to CTL. AHS had significantly higher mRNA abundances of CARD domain containing (*NLRC*)*-3* and *NLRC5* inflammasomes, and lower superoxide dismutase (*SOD*)*-1* and *SOD2* abundance compared with CTL. PF had significantly higher liver weight (% BW) compared to all other groups; while abdominal fat was significantly higher in Pre-CHS compared to CTL, PF, and AHS. Together, these data indicate that the negative effects of HS are partially due to reduced FI. However, the negative effect of HS on gut integrity, average daily gain, feed conversion ratio, and meat yield are direct and independent of the reduced FI during the HS. Thus, warrant investigating the underlying mechanisms in future research.

## Introduction

Global warming is threatening all kinds of life on earth, especially avian species, which are highly susceptible to heat stress (HS) due to the lack of sweat glands and higher core body temperatures compared to mammals (42°C compared to 37°C) (Warren et al., 2018; Emami et al., 2020b). Demand for poultry meat is on the rise and poultry production is expected to increase more than double by 2050 (O’keefe, 2014). However, several factors, including HS, negatively impact the efforts of the poultry industry to meet the high demands by eliciting physiological, behavioral, and production changes in poultry (Emami et al., 2020b; Wasti et al., 2020).

Therefore, evaluating the effects of HS on bird’s physiology, immune response and gut integrity is of substantial importance, and a thorough understanding of these responses is necessary to better design targeted treatments or interventions. The negative effects of acute and cyclic heat stress (AHS and CHS) on performance parameters of broiler chickens is well defined by our research group in previous experiments. During the AHS (2 h) and CHS (3 weeks, 12 h/day at 35°C), feed intake (FI) was significantly reduced in broiler chickens compared to their counterparts maintained under thermoneutral conditions (Flees et al., 2017; Greene et al., 2021a). This, in turn, resulted in a significant reduction in body weight in the CHS birds compared to their thermoneutral counterparts (Greene et al., 2021a). Comparison of four genetically distinct chicken lines with different feed efficiency showed that AHS (2 h) decreased FI in modern broilers (1995 random bred and modern random bred lines) but not in jungle fowl and Athens Canadian random bred chickens (Tabler et al., 2020). Several other studies have shown the negative impacts of HS on broiler chickens’ performance (Del Vesco et al., 2017; Awad et al., 2020).

Heat stress can negatively affect carcass traits as well. Indeed, CHS increased fat deposition, and decreased the proportion of breast muscle, while increasing the proportion of thigh muscle in broiler chickens (Lu et al., 2007; Zhang et al., 2012).

Besides negative effects on performance and carcass traits, the immunosuppressing effects of HS in poultry is well indicated (Lara and Rostagno, 2013). HS causes multiple immune abnormalities in broiler chickens by impairing the developmental process and functional maturation of T- and B-cells in both primary and secondary lymphoid tissues (Hirakawa et al., 2020). Pro-inflammatory cytokines, including interleukin (*IL*)*-1*, *IL2*, *IL6*, *IL18*, and tumor necrosis factor (*TNF*)-α, are involved in the inflammatory response under HS, and excessive pro-inflammatory response may result in tissue damage (Helwig and Leon, 2011; Goel et al., 2021; Greene et al., 2021b). Plasma titers of anti-bovine serum albumin (BSA) immunoglobulin (Ig)Y, IgM, and IgA were lower in broiler chickens exposed to constant HS (35°C) from day 22 to 36 than those of thermoneutral chickens immunized with BSA (Hirakawa et al., 2020). Recently, we have shown that gene expression of circulating inflammatory factors are dysregulated during CHS (Greene et al., 2021b). CHS (12 h/day at 35°C) upregulated the expression of superoxide dismutase (*SOD*)*-1*, *TNF-*α, and C-C motif chemokine ligand (*CCL*)-*4* and *CCL20*; but, downregulated glutathione peroxidase (*GPX*)*-3*, *IL18*, and nucleotide-binding, leucine-rich repeat and pyrin domain containing (*NLRP*)*-3* inflammasome. Heat shock proteins (*HSP*) and nucleotide-binding oligomerization domain, leucine-rich repeat, and CARD domain containing (*NLRC*)*-3* and nucleotide-binding, leucine-rich repeat containing X1 (*NLRX1*) inflammasomes mRNA were unaffected by HS (Greene et al., 2021b).

As mentioned, most of the negative effects of HS on broiler chickens has been attributed to lower FI in heat-stressed birds with no efforts to distinguish the direct and indirect (through reduction in FI) effects of HS on bird’s physiological response. Interestingly, there are indications that the physiological effects of HS are not limited to reduced FI in heat-stressed birds. Heat exposure at 34°C for 15 days significantly increased *IL4* and *IL12* mRNA abundance and decreased interferon (*IFNG*), with no effect on mRNA abundance of *IL6*, *IL10*, *IL13*, and *IL18*. mRNA abundance of *IL4* in the feed-restricted group was higher than that in the control group. Further, *IFNG* abundance increased and *IL12* abundance was not affected by the reduction of FI, suggesting that the FI reduction induced by HS does not modulate splenic cytokine expression in broiler chickens. These data suggest that HS induces spleen involution and affects the expression of splenic cytokines such as *IL12* and *IFNG* in broiler chickens independently of the FI reduction (Ohtsu et al., 2015).

Exposure to HS increased carcass and abdominal fat percentages, and reduced breast, liver and heart percentages which was completely different from pair-fed (PF; feed restricted) chickens that had the lowest fat percentage, and breast percentage similar to birds raised at thermoneutral condition (Zeferino et al., 2016). Recently, researchers indicated that changes in the intestinal morphology and permeability in heat-stressed chickens (24–72 h at 33°C) were due to the HS conditions and not due to the reduced FI (Nanto-Hara et al., 2020). However, the mentioned studies were limited due to addressing a single aspect (such as performance, carcass yield, immune response, or gut integrity) with no measurement of other parameters. In addition, no study has evaluated the effect of CHS and AHS in comparison with feed-restricted birds.

Thus, we conducted this experiment to have a holistic understanding about the physiological changes in broiler chickens during a CHS and distinguish the direct effects of HS from the indirect effects (which are related to the reduction in FI) on broiler chickens’ performance, mortality, and carcass and meat yield. In addition, we evaluated the effect of CHS, AHS, and feed restriction on organs weight, gut integrity and circulating (chemo)cytokines.

## Materials and Methods

### Birds, Diets, and Management

All animal care and procedures were approved by the Institutional Animal Care and Use Committee at the University of Arkansas. On d of hatch, 672 Cobb 500 male broilers were neck tagged, individually weighed, and housed in environmentally controlled chambers in the Poultry Environmental Research Laboratory at the University of Arkansas. There were twelve environmental chambers, each equipped with separate controllers to enable temperature adjustments. Each chamber consisted of two equally sized pens (1.2 × 2.4 m) and all pens were covered with 7 cm pine shavings. Four chambers (eight pens) were allocated to CHS group. Rest of the chambers (eight chambers) were allocated to control (CTL) and PF groups (one pen for each group/chamber) as these groups were raised under the same environmental conditions. Birds were randomly allocated to one of 24 pens with 28 birds/pen. Each pen was equipped with a bucket-type feeder and drinker, and feed and water intake were measured on daily basis from day 0 to 42. Lighting schedule was 24 h light for the first 3 days, reduced to 23 h light:1 h dark day 4 to 7, and reduced further to 18 h light:6 h dark thereafter. Birds were raised in environmentally controlled chambers and temperature and humidity in each pen were recorded every day. Temperature was maintained as follows: 32°C for the first 3 days, then gradually reduced approximately 3°C each week until it reached 23°C on day 21. All birds were fed the same corn-soybean meal basal diet in the form of crumble during the starter (day 0–14), or pellet during the grower (day 15–28) and finisher (day 29–42) period (Table 1). Birds were assigned to one of the three treatments, each with eight replicate pens as follows:

**TABLE 1:** Composition of basal diets (as fed basis, %).

	Period (Days)		
	
Ingredients (%)	Starter (0–14)	Grower (15–28)	Finisher (29–42)
	Crumble	Pellet	Pellet
Corn (7.81% CP)	60.53	60.99	66.52
Soybean meal (48% CP)	32.95	32.55	27.22
Poultry fat (9000 kcal/kg)	1.80	2.38	2.46
Dicalcium phosphate (18.5% P, 22% Ca)	2.08	1.85	1.67
Limestone (37% calcium)	1.10	1.00	0.91
Sodium chloride	0.38	0.40	0.44
DL-methionine (990 g/kg)^1^	0.38	0.30	0.27
L-lysine hydrochloride (788 g L-lysine/kg)^2^	0.37	0.22	0.20
L-threonine (985 g/kg)^3^	0.16	0.08	0.08
Choline chloride (60%)	0.10	0.08	0.08
Vitamin/trace mineral premix^4^	0.15	0.15	0.15
**Calculated analysis (% unless specified)**			
ME (kCal/kg)	2994	3038	3108
Crude protein	21.71	21.30	19.18
Total phosphorus	0.77	0.71	0.66
Available phosphorus	0.45	0.42	0.38
Calcium	0.90	0.84	0.75
Chlorine	0.33	0.32	0.34
Sodium	0.16	0.17	0.19
Potassium	0.84	0.83	0.74
Methionine	0.67	0.59	0.54
Methionine + cysteine	0.98	0.89	0.82
Lysine	1.32	1.18	1.04
Threonine	0.86	0.78	0.70
Linoleic acid	1.46	1.47	1.57
Dietary cation-anion balance	192	196	176

*^1^Rhodimet NP9, ADISSEO, GA, United States.*

*^2^L-Lysine HCl, AJINOMOTO HEARTLAND, INC., Eddyville, IA, United States.*

*^3^FENCHEM Ingredient Technology, Nanjing, China.*

*^4^Vitamins supplied per kg diet: retinol 3.33 mg, cholecalciferol 0.1 mg, α-tocopherol acetate 23.4 mg, vitamin K3 1.2 mg, vitamin B1 1.6 mg, vitamin B2 9.5 mg, niacin 40 mg, pantothenic acid 9.5 mg, vitamin B6 2 mg, folic acid 1 mg, vitamin B12 0.016 mg, biotin 0.05 mg, and choline 556 mg. Minerals supplied per kg diet: Mn 144 mg, Fe 72 mg, Zn 144 mg, Cu 16.2 mg, I 2.1 mg, and Se 0.22 mg.*

1) Control (CTL): birds raised under thermoneutral condition (23°C) from day 29 to 42.
2) CHS: birds exposed to cyclic high ambient temperature (35°C) for 8 h/day (9:30 am to 5:30 pm) from day 29 to 42.
3) Pair-fed (PF): birds raised under thermoneutral condition (23°C) from day 29 to 42, but PF to CHS group, receiving each day 1.05 times the average FI recorded in the HS group in the previous day.

Two days before the CHS, two chickens per pen were randomly selected and a Thermochron temperature logger (iButton, Embedded Data Systems, KY, United States) was placed in the crop *via* oral gavage for continuous monitoring of core body temperature. The environmental temperature and humidity were also continuously recorded in each chamber, inside and outside the barn.

### Performance

On day 0, 28, and 42 birds were weighed individually, while feed and water intake were measured on daily basis. Finally, adjusted average daily gain (ADG), average daily feed intake (ADFI), and feed conversion ratio (FCR) were calculated for day 0–28, day 29–42, and overall experimental period (day 0–42).

### Mortality

Starting at placement, birds were monitored twice daily. For each dead bird, date, neck tag number, body weight, and cause of death were recorded. This procedure continued throughout the study (up to day 42) to record mortality/treatment for each period thus allowing for adjusting performance parameters for daily mortality.

### Carcass and Meat Yield

On day 42, 15 birds/pen (eight pens/treatment) were randomly selected, and wing tagged. Birds were processed using a commercial inline system at the University of Arkansas Pilot Processing Plant (Fayetteville, AR, United States). Birds were electrically stunned (11 V, 11 mA for 11 s), exsanguinated, scaled at 53.8°C for 2 min, and defeathered using a commercial, inline equipment (Foodcraft Model 3; Baker international, MI, United States). Carcasses were manually eviscerated and rinsed before prechilling at 12°C for 15 min. Then, carcasses were chilled for 90 min at 1°C in immersion chilling tanks with manual agitation at 15 min regular intervals. Slaughter weight, and prechill carcasses were recorded, and following a 2 h chill at 4°C, the weight of breasts, tenders, leg quarters, and wings were recorded.

### Gut Integrity, Organ Weights, and Blood Gene Expression Profile

To measure organ weights, gut integrity, and expression of (chemo)cytokines, inflammasomes, *HSP*, and antioxidants in the blood, on day 42 we further divided the birds into five treatments as follow:

1) Control (CTL): birds raised under thermoneutral condition (23°C) from day 29 to 42.
2) AHS: birds raised under thermoneutral condition (23°C) from day 29 to 42, but exposed to 2 h AHS (35°C) before sampling on day 42.
3) Pre-CHS: birds exposed to high ambient temperature (35°C) for 8 h/day (9:30 am–5:30 pm) from day 29 to 42, and sampled before starting the CHS.
4) Post-CHS: birds exposed to high ambient temperature (35°C) for 8 h/day (9:30 am–5:30 pm) from day 29 to 42, and sampled 2 h after starting the CHS.
5) Pair-fed (PF): birds raised under thermoneutral condition (23°C) from day 29 to 42, but PF to CHS group. These birds received each day 1.05 times the average FI recorded in the CHS group in the previous day.

#### Gut Integrity

On day 42, gut integrity was evaluated with the use of fluorescein isothiocyanate-dextran (FITC-d). Two birds/pen from each of the five treatments were selected, and after 1 h fasting, birds were weighed individually, and 8.32 mg/kg FITC-d (4 kDa, Sigma-Aldrich, St. Louis, MO, United States) that were diluted in water were orally gavaged to each bird. Blood was collected 1 h post gavage, and serum collected by centrifugation at 2,000 rpm for 10 min. Sera were diluted 1:5 with PBS, and FITC-d concentration was measured at an excitation wavelength of 485 nm and an emission wavelength of 528 nm (Synergy HT, multimode microplate reader, BioTek Instruments, Inc., Winooski, VT, United States). Pooled sera of three birds from the thermoneutral group that did not receive FITC-d were used for normalization.

#### Organs Weight

On day 42, three birds were selected from each replicate of the five treatments, and liver and abdominal fat weight were recorded, and results were reported as a % of live body weight.

#### RNA Isolation and Quantitative Real-Time PCR

On day 42, 250 μl of whole blood was collected in 750 μl of TRIzol LS (Life Technologies, Carlsbad, CA, United States) from one bird/pen from each of the five treatments, and total RNA was extracted according to the manufacturer’s instruction. cDNA synthesized using qScript cDNA Synthesis Supermix (Quanta Biosciences, Gaithersburg, MD, United States). Finally, target genes (*HSP27*, *HSP60*, *HSP70*, *HSP90*, *GPX1*, *GPX3*, *SOD1*, *SOD2*, *IL6*, *IL10*, *IL18*, *TNF-*α, *CCL4*, *CCL5*, *CCL20*, *NLRC3*, *NLRC5*, *NLRP3*, and *NLRX1*) were amplified using SYBR green master mix (Life Technologies, Carlsbad, CA, United States) and 7500 Real-Time PCR system (Applied Biosystems). Oligonucleotide primer sequences specific for chicken are listed in Table 2. Each reaction was performed in duplicate. Product specificity was confirmed by analysis of the melting curves generated by the 7500 software (version 2.0.3). mRNA abundance was analyzed using tyrosine 3-monooxygenase/tryptophan 5-monooxygenase activation protein zeta (*YWHAZ*) as an endogenous control. Average mRNA abundance relative to *YWHAZ* for each sample was calculated using the 2^–ΔΔCt^ method (Schmittgen and Livak, 2008) with the CTL group as a calibrator.

**TABLE 2:** Oligonucleotide primers for real-time qPCR.

Gene	Accession #	Primer sequence (5′→ 3′)	Orientation	Product size (bp)
*CCL4*	NM_204720.1	CCTGCTGCACCACTTACATAACA	Forward	63
		TGCTGTAGTGCCTCTGGATGA	Reverse	
*CCL5*	NM_001045832.1	TTTCTACACCAGCAGCAAATGC	Forward	59
		GCCCCTTCCTGGTGATGAA	Reverse	
*CCL20*	NM_204438.2	TGCTGCTTGGAGTGAAAATGC	Forward	62
		CAGCAGAGAAGCCAAAATCAAA	Reverse	
*GPX1*	NM_001277853.2	TCCCCTGCAACCAATTCG	Forward	57
		AGCGCAGGATCTCCTCGTT	Reverse	
*GPX3*	NM_001163232.2	GGGCGCTGACCATCGAT	Forward	59
		CATCTTCCCCGCGTACTTTC	Reverse	
*HSP27*	XM_001231557	TTGAAGGCTGGCTCCTGATC	Forward	58
		AAGCCATGCTCATCCATCCT	Reverse	
*HSP60*	NM_001012916	CGCAGACATGCTCCGTTTG	Forward	55
		TCTGGACACCGGCCTGAT	Reverse	
*HSP70*	JO2579	GGGAGAGGGTTGGGCTAGAG	Forward	55
		TTGCCTCCTGCCCAATCA	Reverse	
*HSP90*	X07265.1	TGACCTTGTCAACAATCTTGGTACTAT	Forward	68
		CCTGCAGTGCTTCCATGAAA	Reverse	
*IL6*	NM_204628.1	GCTTCGACGAGGAGAAATGC	Forward	63
		GGTAGGTCTGAAAGGCGAACAG	Reverse	
*IL10*	NM_001004414.2	CGCTGTCACCGCTTCTTCA	Forward	63
		CGTCTCCTTGATCTGCTTGATG	Reverse	
*IL18*	NM_204608.1	TGCAGCTCCAAGGCTTTTAAG	Forward	63
		CTCAAAGGCCAAGAACATTCCT	Reverse	
*NLRC3*	XM_015294675.2	CTCCAACGCCTCACAAACCT	Forward	93
		GCCTTTGGTCATTTCCATCTG	Reverse	
*NLRC5*	NM_001318435.1	CTCGAAGTAGCCCAGCACATT	Forward	80
		CATGTCCAGAGGTGTCAGTCTGA	Reverse	
*NLRP3*	NM_001348947.1	GTTGGGCAGTTTCACAGGAATAG	Forward	63
		GCCGCCTGGTCATACAGTGT	Reverse	
*NLRX1*	XM_004948038.3	GGCTGAAACGTGGCACAAA	Forward	59
		GAGTCCAAGCCCAGAAGACAAG	Reverse	
*SOD1*	NM_205064.1	TGGCTTCCATGTGCATGAAT	Forward	58
		ACGACCTGCGCTGGTACAC	Reverse	
*SOD2*	NM_204211.1	GCTGGAGCCCCACATCAGT	Forward	61
		GGTGGCGTGGTGTTTGCT	Reverse	
*TNF-*α	NM_204267.1	CGTTTGGGAGTGGGCTTTAA	Forward	61
		GCTGATGGCAGAGGCAGAA	Reverse	
*YWHAZ*	NM_001031343.1	ACGCCGTAGGTCATCTTGGA	Forward	58
		ACGGCCTTCCGTCTTTTGT	Reverse	

### Statistical Analyses

Statistical analysis was performed using the one-way ANOVA procedure of JMP software (2015) and significance between treatments (*P* < 0.05) determined by the LSD test. Pen was considered as experimental unit (*n* = 8). The statistical model for data analysis is outlined below:

(1) Yij=μ+Ai+eij

where Yij, measured value for each observation (data); μ, grand mean; Ai, treatment effect; and eij, experimental error.

## Results

### Core Body Temperature

**FIGURE 1:**
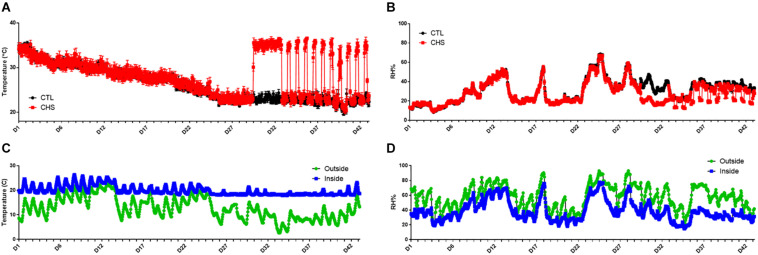
Temperature and relative humidity (RH) fluctuation in the environmental chambers **(A,B)**, and inside and outside the barn **(C,D)** during the cyclic heat stress (CHS) experiment. Birds raised under recommended conditions from day 0 to 28. From day 29 to 42, birds either raised at thermoneutral temperature (CTL, 23°C) or exposed to CHS (35°C). Data are presented as mean ± SEM (*n* = 1/chamber or 8/group).

**FIGURE 2:**
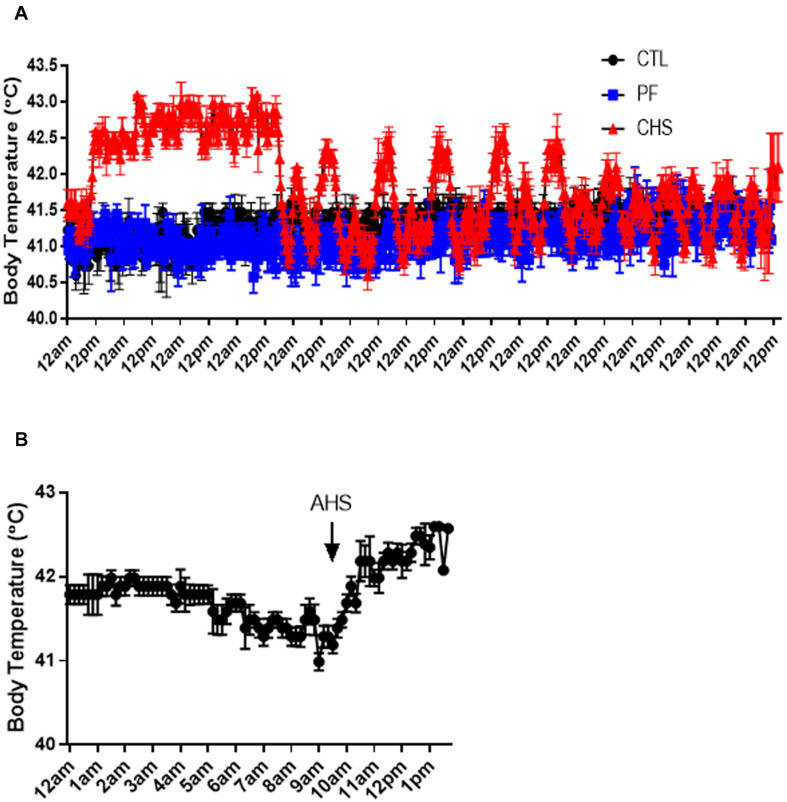
Core body temperature fluctuation during heat stress (HS). **(A)** Treatments include: (1) control (CTL): birds raised under normal temperature (23°C) from day 29 to 42 and had free access to the diet; (2) cyclic heat stress (CHS): birds exposed to high temperatures (8 h/day at 35°C; from 9:30 am to 5:30 pm) from day 29 to 42 and had free access to the diet; (3) pair-fed (PF): birds raised under the same condition as CTL group, but fed the same amount of feed as CHS group from day 29 to 42. **(B)** Core body temperature fluctuation during an acute heat stress (AHS). Birds in this group raised under thermoneutral condition (23°C) from day 29 to 42 but exposed to 2 h AHS (35°C) before sampling on day 42. Data are presented as mean ± SEM (*n* = 8; two birds/pen).

Core body temperature, inside chamber as well as environmental (inside and outside barn) temperature and relative humidity are shown in Figures 1, 2. On average, core body temperature was approximately 1°C higher in CHS group compared to CTL and PF groups (Figure 2).

### Mortality

Mortality data is presented in Table 3. Mortality was low in all the treatments during the starter, grower, finisher, and overall experimental period and there was no difference between treatments.

**TABLE 3:** Effect of rearing temperature and feed restriction on mortality (%) of Cobb 500 male broiler chickens.

Treatments^1^	Time period (day)		
	
	0–28	29–42	0–42
CTL	0.89	1.34	2.23
CHS	3.12	0.93	4.01
PF	1.78	0.46	2.23
SEM^2^	0.96	0.58	1.12
*P*-value^3^	0.282	0.573	0.444

*^1^Treatments include: (1) control (CTL): birds raised under normal temperature (23°C) from day 29 to 42 and had free access to the diet; (2) cyclic heat stress (CHS): birds exposed to high temperatures (8 h/day at 35°C; from 9:30 am–5:30 pm) from day 29 to 42 and had free access to the diet; (3) pair-fed (PF): birds raised under the same condition as CTL group, but fed the same amount of feed as CHS group from day 29 to 42. All birds fed the same corn-soybean meal basal diet.*

*^2^SEM: pooled standard error of means.*

*^3^*P*-value: based on one-way ANOVA.*

### Performance

Table 4 indicates performance parameters during various phases of the experiment. Before the start of CHS (day 0–28) performance including BW, ADFI, ADG, and FCR was similar for all the treatments. However, for the finisher phase (day 29–42), birds in the CTL group had significantly higher ADFI, and ADG compared to CHS and PF groups. In addition, PF birds had higher ADG compared to the CHS group. FCR was significantly better for both CTL and PF groups compared to CHS, and there was no difference in FCR for CTL and PF groups. Differences in the finisher phase led to significant difference in performance among the treatments during the overall experimental period. Birds in the CTL group had the highest ADFI, and ADG which was significantly higher than CHS and PF groups. Likewise, PF birds had significantly higher ADG compared to CHS, despite the same ADFI. Birds in the CTL and PF group had similar FCR which was significantly better that the CHS group.

**TABLE 4:** Effect of rearing temperature and feed restriction on Cobb 500 male broiler chicken’s performance.^1^

	Treatments^2^			Statistics	
		
Item^3^	CTL	CHS	PF	SEM^4^	*P*-value^5^
**Body weight (g)**					
Day 0	38.37	38.28	38.59	0.19	0.523
Day 28	1839.54	1812.25	1828.44	17.16	0.537
Day 42	3701.42	3215.29	3381.62	28.88	<0.001

**Day 0–28**					
ADFI, g	87.57	87.47	87.66	0.71	0.982
ADG, g	64.32	63.35	63.92	0.61	0.537
FCR	1.36	1.38	1.37	0.01	0.112
**Day 29–42**					
ADFI, g	216.09^a^	181.95^b^	183.67^b^	2.31	<0.001
ADG, g	132.99^a^	100.68^c^	110.03^b^	1.86	<0.001
FCR	1.62^b^	1.81^a^	1.66^b^	0.02	<0.001
**Day 0–42**					
ADFI, g	128.32^a^	117.43^b^	118.10^b^	0.91	<0.001
ADG, g	87.21^a^	75.79^c^	79.29^b^	0.71	<0.001
FCR	1.47^b^	1.55^a^	1.48^b^	0.01	<0.001

*^*a*–*c*^In each row, means with different letters are significantly different (*P* < 0.05).*

*^1^Data represent the mean value of eight replicate pens of 28 birds/pen (*n* = 8).*

*^2^Treatments include: (1) control (CTL): birds raised under normal temperature (23°C) from day 29 to 42 and had free access to the diet; (2) cyclic heat stress (CHS): birds exposed to high temperatures (8 h/day at 35°C; from 9:30 am to 5:30 pm) from day 29 to 42 and had free access to the diet; (3) pair-fed (PF): birds raised under the same condition as CTL group, but fed the same amount of feed as CHS group from day 29 to 42.*

*^3^ADFI, average daily feed intake; ADG, average daily gain; FCR, feed conversion ratio.*

*^4^SEM: pooled standard error of means.*

*^5^*P*-value: based on one-way ANOVA.*

### Carcass and Meat Yield

Carcass and meat yield is shown in Table 5. Hot carcass weight (% live body weight), was significantly higher in the CTL and CHS compared to PF group. Breast yield (% hot carcass weight) was significantly higher in CTL compared to CHS and PF. In addition, PF group had significantly higher breast yield compared to CHS group. On the contrary, leg quarters and wings (% hot carcass weight) were significantly higher in CHS compared to CTL and PF groups. Furthermore, tender yield (% hot carcass weight) was higher in CHS compared to CTL but not PF group.

**TABLE 5:** Effect of rearing temperature and feed restriction on carcass and meat yield of Cobb 500 male broiler chickens on day 42.^1^

Treatments^2^	Hot carcass (% body weight)	Meat yield (% hot carcass weight)			
		
		Breast	Tender	Leg quarters	Wings
CTL	71.99^a^	31.07^a^	5.05^b^	28.84^c^	10.42^b^
CHS	71.37^a^	29.18^c^	5.24^a^	30.44^a^	10.71^a^
PF	70.60^b^	29.99^b^	5.12^ab^	29.72^b^	10.37^b^
SEM^3^	0.25	0.17	0.05	0.13	0.05
*P*-value^4^	0.003	<0.001	0.036	<0.001	<0.001

*^*a*–*c*^In each column, means with different letters are significantly different (*P* < 0.05).*

*^1^Data represent the mean value of eight replicate pens of 15 birds/pen (*n* = 8).*

*^2^Treatments include: (1) control (CTL): birds raised under normal temperature (23°C) from day 29 to 42 and had free access to the diet; (2) cyclic heat stress (CHS): birds exposed to high temperatures (8 h/day at 35°C; from 9:30 am to 5:30 pm) from day 29 to 42 and had free access to the diet; (3) pair-fed (PF): birds raised under the same condition as CTL group, but fed the same amount of feed as CHS group from day 29 to 42.*

*^3^SEM: pooled standard error of means.*

*^4^*P*-value: based on one-way ANOVA.*

### Organs Weight

Table 6 indicates liver and abdominal fat (including fat surrounding gizzard, proventriculus, bursa of fabricius, cloaca, and adjacent muscles) as a % of live body weight in different groups. Birds in the PF group had the highest liver weight compared to all other groups. On the contrary, Post-CHS and AHS birds had the lowest liver weight that was significantly different from Pre-CHS and CTL groups. Pre-CHS had the highest abdominal fat which was significantly different from PF and AHS groups.

**TABLE 6:** Effect of temperature and feed restriction on organ weight of Cobb 500 male broiler chickens on day 42.^1^

Treatments^2^	Organ weight (% body weight)	
	
	Liver	Abdominal fat^3^
CTL	1.75^b^	1.02^ab^
PF	1.94^a^	0.96^b^
Pre-CHS	1.75^b^	1.14^a^
Post-CHS	1.44^c^	1.04^ab^
AHS	1.53^c^	0.96^b^
SEM^4^	0.05	0.05
*P*-value^5^	<0.001	0.048

*^*a*–*c*^In each column, means with different letters are significantly different (*P* < 0.05).*

*^1^Data represent the mean value of eight replicate pens of three birds/pen (*n* = 8).*

*^2^Treatments include: (1) control (CTL): birds raised under normal temperature (23°C) from day 29 to 42 and had free access to the diet; (2) acute heat stress (AHS): birds from CTL group after a 2 h heat exposure on day 42 (35°C); (3 and 4) cyclic heat stress (CHS): birds exposed to high temperatures (8 h/day at 35°C; from 9:30 am to 5:30 pm) from day 29 to 42 and sampled before the start (Pre-CHS) or 2 h after the start of CHS (Post-CHS) on day 42; (5) pair-fed (PF): birds raised under the same temperature as CTL group, but fed the same amount of feed as CHS group from day 29 to 42.*

*^3^Fat surrounding gizzard, proventriculus, bursa of fabricius, cloaca, and adjacent muscles.*

*^4^SEM: pooled standard error of means.*

*^5^*P*-value: based on one-way ANOVA.*

### Gut Integrity

**FIGURE 3:**
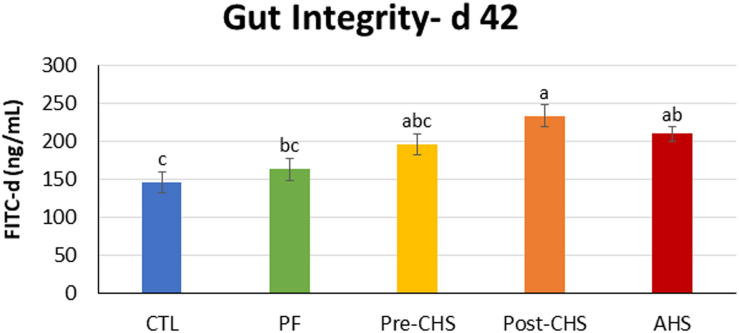
FITC-d concentration in the serum of Cobb 500 male broiler chickens on day 42. Birds in all the groups were raised under the recommended conditions from day 0 to 28. On day 42, birds fasted for 2 h, then individually weighed and orally gavage with 8.32 mg FITC-d/kg body weight, and blood was collected 2 h post gavage. Treatments include: (1) control (CTL): birds raised under thermoneutral condition (23°C) from day 29 to 42; (2) AHS: birds raised under thermoneutral condition (23°C) from day 29 to 42, but exposed to 2 h AHS (35°C) before sampling on day 42; (3) Pre-CHS: birds exposed to high ambient temperature (35°C) for 8 h/day (9:30 am–5:30 pm) from day 29 to 42, and sampled before starting the CHS; (4) Post-CHS: birds exposed to high ambient temperature (35°C) for 8 h/day (9:30 am–5:30 pm) from day 29 to 42, and sampled 2 h after starting the CHS; (5) pair-fed (PF): birds raised under thermoneutral condition (23°C) from day 29 to 42, but pair-fed to CHS group (these birds received each day 1.05 times the average feed intake recorded in the CHS group in the previous day). Results are given as means (*n* = 8; two birds/pen) for each group. Error bars indicate standard errors. Columns with different letters are significantly different (*P* < 0.05).

Figure 3 shows the FITC-d concentration in the sera of different groups. There was a significant difference in the concentration of FITC-d in Post-CHS and AHS groups compared to the CTL. Furthermore, Post-CHS group had higher FITC-d concentration in the sera (lower gut integrity) compared to the PF group.

### Blood (Chemo)Cytokines, Inflammasomes, Heat Shock Proteins, and Antioxidants

**FIGURE 4:**
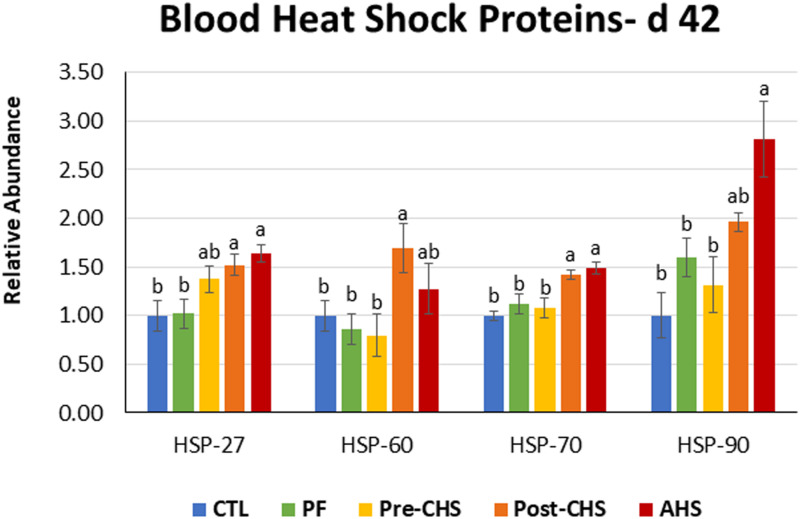
Relative mRNA abundance of heat shock protein (*HSP*)-*27*, *HSP60*, *HSP70*, and *HSP90* in the blood of Cobb 500 male broiler chickens on day 42. Birds in all the groups were raised under the recommended conditions from day 0 to 28. Treatments include: (1) control (CTL): birds raised under thermoneutral condition (23°C) from day 29 to 42; (2) AHS: birds raised under thermoneutral condition (23°C) from day 29 to 42, but exposed to 2 h AHS (35°C) before sampling on day 42; (3) Pre-CHS: birds exposed to high ambient temperature (35°C) for 8 h/day (9:30 am–5:30 pm) from day 29 to 42, and sampled before starting the CHS; (4) Post-CHS: birds exposed to high ambient temperature (35°C) for 8 h/day (9:30 am–5:30 pm) from day 29 to 42, and sampled 2 h after starting the CHS; (5) pair-fed (PF): birds raised under thermoneutral condition (23°C) from day 29 to 42, but pair-fed to CHS group (these birds received each day 1.05 times the average feed intake recorded in the CHS group in the previous day). Values are represented as a *n*-fold difference relative to the calibrator (CTL). Results are given as means (*n* = 8) for each group. Error bars indicate standard errors. For each gene, bars with different letters are significantly different (*P* < 0.05).

**FIGURE 5:**
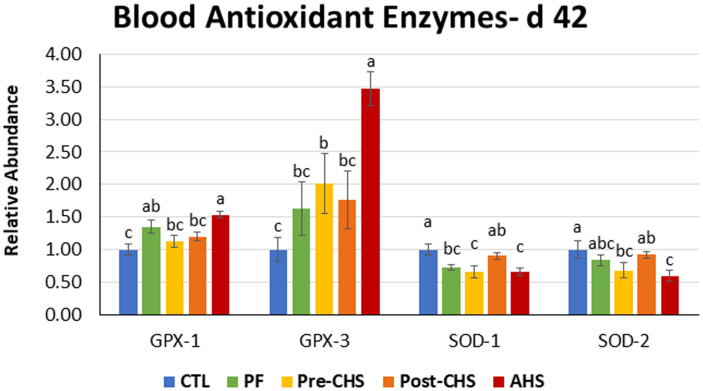
Relative mRNA abundance of superoxide dismutase (*SOD*)-*1*, *SOD2*, glutathione peroxidase (*GPX*)-*1*, and *GPX2* in the blood of Cobb 500 male broiler chickens on day 42. Birds in all the groups were raised under the recommended conditions from day 0 to 28. Treatments include: (1) control (CTL): birds raised under thermoneutral condition (23°C) from day 29 to 42; (2) AHS: birds raised under thermoneutral condition (23°C) from day 29 to 42, but exposed to 2 h AHS (35°C) before sampling on day 42; (3) Pre-CHS: birds exposed to high ambient temperature (35°C) for 8 h/day (9:30 am–5:30 pm) from day 29 to 42, and sampled before starting the CHS; (4) Post-CHS: birds exposed to high ambient temperature (35°C) for 8 h/day (9:30 am–5:30 pm) from day 29 to 42, and sampled 2 h after starting the CHS; (5) pair-fed (PF): birds raised under thermoneutral condition (23°C) from day 29 to 42, but pair-fed to CHS group (these birds received each day 1.05 times the average feed intake recorded in the CHS group in the previous day). Values are represented as a *n-*fold difference relative to the calibrator (CTL). Results are given as means (*n* = 8) for each group. Error bars indicate standard errors. For each gene, bars with different letters are significantly different (*P* < 0.05).

**FIGURE 6:**
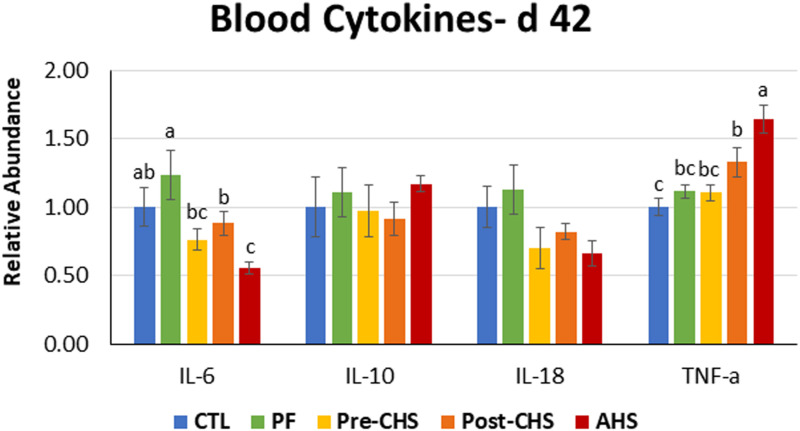
Relative mRNA abundance of interleukin (*IL*)-*6*, *IL10*, *IL18*, and tumor necrosis factor-α in the blood of Cobb 500 male broiler chickens on day 42. Birds in all the groups were raised under the recommended conditions from day 0 to 28. Treatments include: (1) control (CTL): birds raised under thermoneutral condition (23°C) from day 29 to 42; (2) AHS: birds raised under thermoneutral condition (23°C) from day 29 to 42, but exposed to 2 h AHS (35°C) before sampling on day 42; (3) Pre-CHS: birds exposed to high ambient temperature (35°C) for 8 h/day (9:30 am–5:30 pm) from day 29 to 42, and sampled before starting the CHS; (4) Post-CHS: birds exposed to high ambient temperature (35°C) for 8 h/day (9:30 am–5:30 pm) from day 29 to 42, and sampled 2 h after starting the CHS; (5) pair-fed (PF): birds raised under thermoneutral condition (23°C) from day 29 to 42, but pair-fed to CHS group (these birds received each day 1.05 times the average feed intake recorded in the CHS group in the previous day). Values are represented as a *n*-fold difference relative to the calibrator (CTL). Results are given as means (*n* = 8) for each group. Error bars indicate standard errors. For each gene, bars with different letters are significantly different (*P* < 0.05).

**FIGURE 7:**
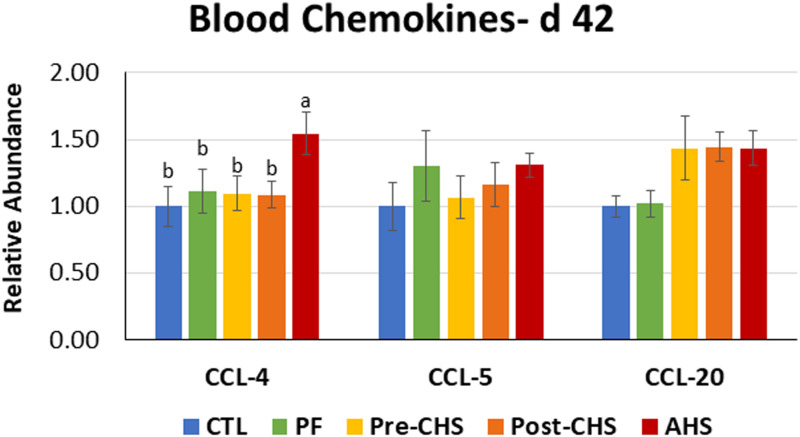
Relative mRNA abundance of C-C motif chemokine ligand (*CCL*)-*4*, *CCL5*, and *CCL20* in the blood of Cobb 500 male broiler chickens on day 42. Birds in all the groups were raised under the recommended conditions from day 0 to 28. Treatments include: (1) control (CTL): birds raised under thermoneutral condition (23°C) from day 29 to 42; (2) AHS: birds raised under thermoneutral condition (23°C) from day 29 to 42, but exposed to 2 h AHS (35°C) before sampling on day 42; (3) Pre-CHS: birds exposed to high ambient temperature (35°C) for 8 h/day (9:30 am–5:30 pm) from day 29 to 42, and sampled before starting the CHS; (4) Post-CHS: birds exposed to high ambient temperature (35°C) for 8 h/day (9:30 am–5:30 pm) from day 29 to 42, and sampled 2 h after starting the CHS; (5) pair-fed (PF): birds raised under thermoneutral condition (23°C) from day 29 to 42, but pair-fed to CHS group (these birds received each day 1.05 times the average feed intake recorded in the CHS group in the previous day). Values are represented as a *n*-fold difference relative to the calibrator (CTL). Results are given as means (*n* = 8) for each group. Error bars indicate standard errors. For each gene, bars with different letters are significantly different (*P* < 0.05).

**FIGURE 8:**
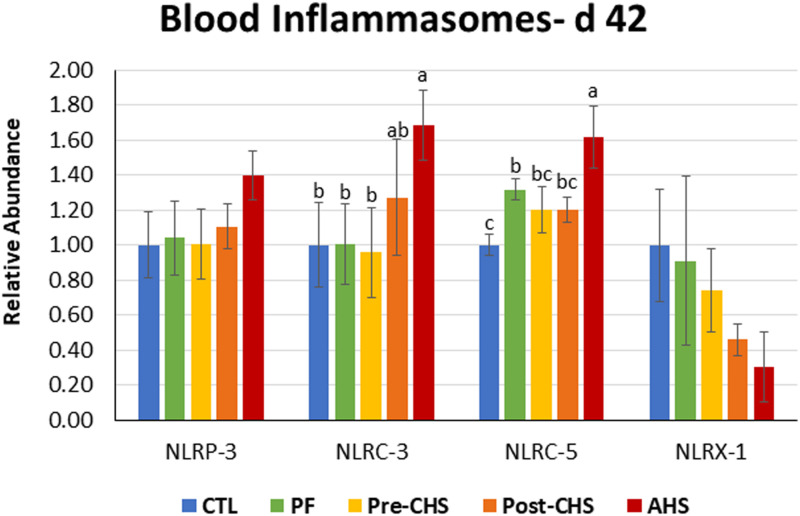
Relative mRNA abundance of nucleotide-binding, leucine-rich repeat and pyrin domain containing (*NLRP*)-*3*, nucleotide-binding oligomerization domain, leucine-rich repeat, and CARD domain containing (*NLRC*)-*3*, *NLRC5*, and nucleotide-binding, leucine-rich repeat containing X1 (NLRX1) in the blood of Cobb 500 male broiler chickens on day 42. Birds in all the groups were raised under the recommended conditions from day 0 to 28. Treatments include: (1) control (CTL): birds raised under thermoneutral condition (23°C) from day 29 to 42; (2) AHS: birds raised under thermoneutral condition (23°C) from day 29 to 42, but exposed to 2 h AHS (35°C) before sampling on day 42; (3) Pre-CHS: birds exposed to high ambient temperature (35°C) for 8 h/day (9:30 am–5:30 pm) from day 29 to 42, and sampled before starting the CHS; (4) Post-CHS: birds exposed to high ambient temperature (35°C) for 8 h/day (9:30 am–5:30 pm) from day 29 to 42, and sampled 2 h after starting the CHS; (5) pair-fed (PF): birds raised under thermoneutral condition (23°C) from day 29 to 42, but pair-fed to CHS group (these birds received each day 1.05 times the average feed intake recorded in the CHS group in the previous day). Values are represented as a *n*-fold difference relative to the calibrator (CTL). Results are given as means (*n* = 8) for each group. Error bars indicate standard errors. For each gene, bars with different letters are significantly different (*P* < 0.05).

mRNA abundances of HSPs, antioxidant enzymes, cytokines, chemokines, and inflammasomes are shown in Figures 4–8, respectively. *HSP27* abundance was significantly higher in Post-CHS and AHS compared CTL and PF. Post-CHS was the only group with significantly higher abundance of *HSP60* compared to CTL, PF and Pre-CHS; while, *HSP90* was significantly higher in the AHS group compared to CTL, PF and Pre-CHS. Both Post-CHS and AHS had significantly higher *HSP70* mRNA abundance in the circulation compared to other groups (Figure 4).

Control group had significantly lower *GPX1* abundance compared to PF and AHS, while *GPX3* was significantly lower in the CTL group compared to Pre-CHS and AHS. AHS significantly upregulated mRNA abundance for *GPX1* compared to other groups except PF. In addition, AHS group had the highest *GPX3* abundance that were significantly different from all other groups. On the contrary, mRNA abundance of *SOD1* was significantly lower in PF, Pre-CHS, and AHS compared to CTL. In addition, mRNA abundance of *SOD2* was significantly lower in Pre-CHS, and AHS compared to CTL group (Figure 5).

mRNA abundances of *IL10* and *IL18* in the circulation showed no difference among the treatments. *IL6* abundance was significantly lower in AHS compared to CTL and PF; while, *IL6* abundance in Pre-CHS and Post-CHS was significantly lower compared to PF. In addition, mRNA abundance of TNF-α was significantly higher in AHS group compared to all other groups. However, for the Post-CHS group, mRNA abundance of TNF-α was significantly higher only compared to the CTL (Figure 6).

Chemokine expression profile showed no differences in mRNA abundance of *CCL5*, and *CCL20* in the blood samples from different groups. However, birds in the AHS group had significantly higher abundance of *CCL4* compared to other groups (Figure 7).

Circulatory inflammasomes including *NLRP3* and *NLRX1* abundance was not affected by any of the treatment groups. However, mRNA abundance of *NLRC3* was significantly higher in the AHS group compared to CTL, PF, and Pre-CHS groups. In addition, mRNA abundance of *NLRC5* was significantly higher in AHS compared to other groups. Interestingly, *NLRC5* abundance was higher in PF group compared to the CTL (Figure 8).

## Discussion

Birds raised under thermoneutral condition had significantly higher ADG, and ADFI compared to CHS and PF groups. Previously, researchers including our group reported that both chronic and constant HS reduced ADG and ADFI, and increased FCR compared to birds raised under thermoneutral condition (Zeferino et al., 2016; Baxter et al., 2020; Tabler et al., 2020; Greene et al., 2021a). Similarly, heat exposure (6 h/day at 34°C from day 22 to 35) significantly reduced FI and body weight gain (day 22–35 and 1–35), while increased FCR (day 22–35 and 1–35) and serum levels of acute phase proteins (APPs) in both Cobb and Ross broiler chickens (Awad et al., 2020).

Despite similar ADFI, PF group had significantly higher ADG compared to CHS group in the current experiment, which is different from the previous study where growth performance was similar between PF and heat-stressed chickens (Zeferino et al., 2016). These inconsistencies might be due to differences in HS regimes (CHS in our study vs. constant HS) or type of housing (floor pens in our study vs. cages) among trials. Lower ADG in CHS compared to PF group in our experiment might be justified by the higher mRNA abundance of HSP’s in the circulation, and disrupted gut integrity. This might be indicative of nutrient allocation toward maintaining gut integrity and homeostasis instead of growth in order to deal with the negative impacts of the CHS, and brings the theory of resource allocation into attention (Siegel and Honaker, 2009). The impact of HS on the inflammatory response in poultry may be a significant contributor to the reductions seen in growth and productivity (Lara and Rostagno, 2013). HS causes multiple immune abnormalities in broiler chickens by impairing the developmental process and functional maturation of T- and B-cells in both primary and secondary lymphoid tissues, and increase the expression of pro-inflammatory cytokines, presumably through an enhanced proliferation of lymphocytes and macrophages (Hirakawa et al., 2020; Goel et al., 2021). Broilers subjected to HS had lower levels of total circulating antibodies, as well as lower specific IgM and IgG levels, both during primary and secondary humoral responses (Bartlett and Smith, 2003). HS- induced decrease in the number of lymphocytes in the cortex and medulla areas of the bursa was reported (Aengwanich, 2008). Pro-inflammatory cytokine family, including interleukin *IL1*, *IL2*, *IL6*, *IL18*, and *TNF-*α, have been shown to play an active role in the inflammatory response under high ambient temperature (Helwig and Leon, 2011). HS (31°C from day 35 to 42) did not affect mRNA abundance of *IL1B*, *IL6*, *IL10*, *IL12*, *IL13*, *IFNG* in the spleen and cecal tonsils of broiler chickens; However, it increased mRNA abundance of *TGFB* in the cecal tonsils, with no effect on mRNA abundance of *TGFB* in spleen (Quinteiro-Filho et al., 2017). The mRNA abundance of *IL1B* significantly decreased in the spleen of Three-yellow chicken during AHS, whereas it was not affected in the Kirin chicken. The mRNA abundance of *IL6* and *IL10* were increased in the spleen of Kirin chicken subjected to AHS, but their abundance was not affected in Three-yellow chicken due to the AHS (Adu-Asiamah et al., 2021). AHS (7 h at 40°C) significantly decreased *IFNB*, and *IL4* concentration in the plasma of broiler chickens (Saleh and Al-Zghoul, 2019).

Dysregulation of circulating inflammatory factors during CHS was previously reported by our group which is not in line with the results of the current study. Compared to birds raised under thermoneutral condition, CHS (12 h/day; 35°C) increased mRNA abundance of *SOD1*, *TNF-*α, *CCL4*, and *CCL20*, downregulated *GPX3*, *IL18*, and *NLRP3* inflammasome, with no effect on HSPs, *NLRC3* and *NLRX1* inflammasomes (Greene et al., 2021b). Discrepancies might be due to the duration of HS (8 vs. 12 h/day).

Regulated gut integrity is essential for controlling the transfer of pathogenic bacteria and immunogens and is considered a very essential and critical checkpoint for the regulation of immune responses (Emami et al., 2019, 2020a). Thus, altered gut integrity in the CHS group but not in the PF, confirm that the negative effects of CHS cannot be attributed only to the lower FI. Similarly, others showed that the changes observed in the intestinal permeability in chickens subjected to HS for 24–72 h are due to the HS conditions and not due to reduced FI (Nanto-Hara et al., 2020). In addition, results of current experiment showed that CHS and AHS both had a negative impact on the gut integrity, suggesting the rapid onset and continuation of leaky gut during the HS.

Birds in the CTL group had significantly higher hot carcass yield compared to PF but not CHS group. In contrast, HS reduced slaughter and carcass weights compared to birds raised under thermoneutral condition (Zeferino et al., 2016; Greene et al., 2021a). Despite the lowest ADG and final live body weight, birds in the CHS group had higher hot carcass yield compared to the PF group on day 42. Others reported increased carcass and abdominal fat percentages in broilers exposed to HS compared to their PF counterparts (Zeferino et al., 2016). Higher carcass yield in the CHS compared to the PF might be due to significantly lower liver weight (in %) compared to PF group. Previous studies confirm that birds exposed to HS had lower internal organ weights including thymus, bursa, spleen, liver, and heart (Bartlett and Smith, 2003; Zeferino et al., 2016). Furthermore, reduced liver weight was reported in laying hens subjected to CHS conditions (Felver-Gant et al., 2012). Besides lower organs weight, higher hot carcass yield in the CHS group might be due to higher leg quarters and wing% in the CHS group compared to PF.

Cyclic heat stress exposure decreased breast yield, which was significantly lower than that in CTL and PF groups. It has been reported that the effects of CHS is breed-dependent and HS negatively affects breast yield in fast growing broilers (arbor acres), but not slow growing chickens (Beijing You Chicken) (Lu et al., 2007). Similarly, CHS decreased the proportion of breast muscle, while increasing the proportion of thigh muscle and fat deposition in broilers (Zhang et al., 2012). Previously, we showed the negative effects of CHS on breast weight (Greene et al., 2021a). Exposure to HS reduced breast %; however, in contrast to the results of our experiment, PF chickens’ breast % was similar to thermoneutral raised birds (Zeferino et al., 2016). Higher leg quarters and wings %, despite lower breast yield due to CHS might be due to the allocation of resources to the muscles that are involved in the movement (legs and wings), rather than breast which is mostly considered as a storage. On the top of that, blood which contains the nutrients might have been directed toward the limbs (extremities) as a mechanism for heat dissipation.

As mentioned, PF group had the highest liver % which was significantly higher compared to other groups, while abdominal fat was significantly higher in birds exposed to CHS. Lower abdominal fat % in the PF group compared to the birds raised under CHS was previously reported by other researchers (Zeferino et al., 2016). This brings an interesting hypothesis into consideration which is the distinct regulatory mechanisms of energy metabolism in the body of PF vs. CHS birds. With this regard, it seems that the energy metabolism in PF group leans toward glycogen metabolism (short-term); while, in the CHS birds the regulation of energy metabolism seems to be directed toward the fat metabolism (long-term). Further consideration, assessment and comparison of the key regulators of lipogenesis, lipolysis, and glycogenesis in the liver and fat tissue of CHS compared to the PF group is warranted.

## Conclusion

Chronic CHS exerts its negative effects on performance, and body composition of broiler chickens through direct mechanisms that are independent of FI depression. Additionally, CHS and AHS both exert negative effects on broiler chickens’ gut integrity. However, at the mRNA level, only AHS dysregulate inflammatory responses in the circulation which suggests that the involved mechanisms occur rapidly during the HS.

## Data Availability Statement

The raw data supporting the conclusions of this article will be made available by the authors, without undue reservation.

## Ethics Statement

All animal care and procedures were approved by the Institutional Animal Care and Use Committee at the University of Arkansas.

## Author Contributions

SD conceptualized and designed the experiment. NE, EG, and SD performed the experiment. NE wrote the first draft. SD edited the manuscript with a critical review by MK. All authors contributed to the article and approved the submitted version.

## Conflict of Interest

The authors declare that the research was conducted in the absence of any commercial or financial relationships that could be construed as a potential conflict of interest.
